# Strategies to Tune Electrospun Scaffold Porosity for Effective Cell Response in Tissue Engineering

**DOI:** 10.3390/jfb10030030

**Published:** 2019-07-09

**Authors:** Jimna Mohamed Ameer, Anil Kumar PR, Naresh Kasoju

**Affiliations:** Division of Tissue Culture, Department of Applied Biology, Biomedical Technology Wing, Sree Chitra Tirunal Institute for Medical Sciences and Technology, Thiruvananthapuram 695012, Kerala, India

**Keywords:** sacrificial fibers, salt leaching, gas foaming, electrospray, ultrasonication, liquid bath collector, anisotropic pores, air impedance, laser ablation, 3D printing

## Abstract

Tissue engineering aims to develop artificial human tissues by culturing cells on a scaffold in the presence of biochemical cues. Properties of scaffold such as architecture and composition highly influence the overall cell response. Electrospinning has emerged as one of the most affordable, versatile, and successful approaches to develop nonwoven nano/microscale fibrous scaffolds whose structural features resemble that of the native extracellular matrix. However, dense packing of the fibers leads to small-sized pores which obstruct cell infiltration and therefore is a major limitation for their use in tissue engineering applications. To this end, a variety of approaches have been investigated to enhance the pore properties of the electrospun scaffolds. In this review, we collect state-of-the-art modification methods and summarize them into six classes as follows: approaches focused on optimization of packing density by (a) conventional setup, (b) sequential or co-electrospinning setups, (c) involving sacrificial elements, (d) using special collectors, (e) post-production processing, and (f) other specialized methods. Overall, this review covers historical as well as latest methodologies in the field and therefore acts as a quick reference for those interested in electrospinning matrices for tissue engineering and beyond.

## 1. Introduction

**Figure 1:**
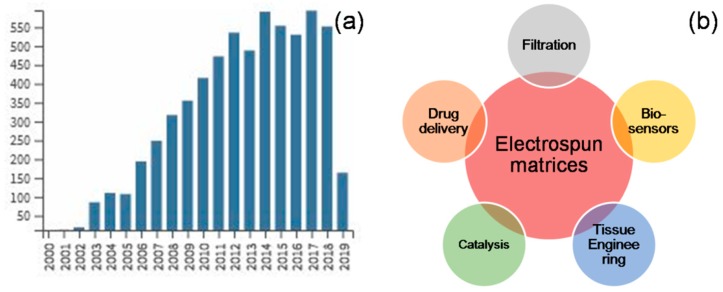
Trends in electrospinning: Number of publications on electrospinning has seen an exponential increase in recent years (**a**), and the areas of applications have been diverse across various disciplines (**b**) (publication trend was obtained from WoS Core Collection, search string “electrospinning” in “Title”, time span “1984–2019”).

With the emergence of nanotechnology, researchers are more interested in developing and studying nano- and sub-micron scale fibrous materials for a variety of applications [1]. Amongst an array of fabrication techniques such as bi-component extrusion, drawing, phase separation, template synthesis, self-assembly, meltblown technology, centrifugal spinning, and electrospinning, the latter method has gained attention of many as a simple, cost-effective, versatile voltage-driven process to produce fibers ranging in diameter from a few microns down to tens of nanometer [2]. William Gilbert observed the deformation of a liquid droplet as electrically charged amber piece was brought near the droplet [3]. This eventually came to know as ‘Taylor cone’ and this was the first recorded observation of electrospraying. The amount of charge required to deform a droplet was observed by Rayleigh in 1897 and the experiment set for production of artificial fibers was patented by Formhals in 1934 [4]. Formhals also published a series of patents, describing an experimental setup for the production of polymer fibers using an electrostatic force [4,5,6,7,8], whereas the work of Geoffrey Ingram Taylor (1969) on electrically driven jets has laid the groundwork for electrospinning [9]. Worldwide research institutes are studying various aspects of the electrospinning process and the number of publications based on electrospinning has grown in recent years (Figure 1).

**Figure 2:**
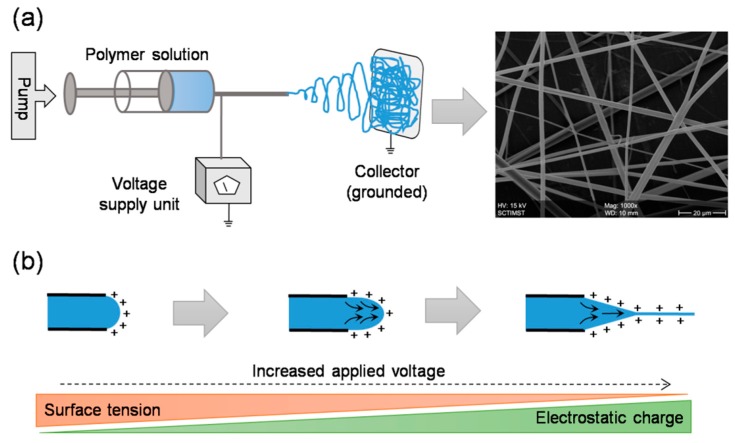
Electrospinning setup and working principle: A typical setup includes a syringe pump, a syringe with blunt end needle, a high voltage power supply, and a grounded collector (**a**). In principle, upon applying high voltage, the electrostatic repulsions dominate the surface tension and lead to the formation of a smooth continuous jet (**b**). Before the jet reaches the grounded collector, the solvent evaporates and thus results in the formation of fibrous network.

The basic setup involves a syringe with blunt-ended needle loaded with polymer solution of interest, a syringe pump which drives the solution at a steady rate, a high voltage power source connected to the needle, and a grounded collector placed in a defined distance from needle tip (Figure 2) [10]. While the solution is pumped out at a specific flow rate, a droplet of the solution comes out at the spinneret. Upon applying high voltage, the charge gets accumulated at the droplet surface and consequently the electrostatic repulsions dominate the surface tension, thereby, the liquid meniscus is deformed into a conical shaped structure known as Taylor cone and leads to the ejection of fluid jet from the tip of the cone. The charged jet elongates, becomes thin, and dry as it travels and deposits on a grounded collector [11]. Alternatively, needleless setup was also introduced to enable multiple spinnerets and thereby to scale up the fiber production [12]. Both approaches have been explored in different contexts and the resultant matrices were used in diverse fields [13]. For instance, they have been explored as filters for volatile organic compounds, nanoparticles, and airborne bacterial contaminates [14], as substrates for enzyme immobilization in fine chemistry, biomedicine, and biosensor [15,16], and as scaffolds for cell adhesion and growth in tissue engineering and regenerative medicine [17,18].

In this review, we begin, in Section 2, with a brief background of tissue engineering scaffolds, features of native extracellular matrix (ECM) and importance of mimicking these features in scaffolds for successful tissue regeneration, electrospun matrices as biomimetic scaffolds in engineering various tissues with few examples, limitations in electrospun scaffolds that are associated with high dense packing of fibers and its consequences on cell infiltration. This is followed by, in Section 3, a detailed account of various strategies to enhance electrospun scaffold porosity in order to enhance cell infiltration and subsequent tissue regeneration. In particular, this section highlights the state-of-the-art methods that we attempted to categorize into six broad classes. First category describes the approaches that are based on systematic optimization of electrospinning parameters without making any alterations to the experimental setup (Section 3.1). Second category describes the use of two or more electrospinning setups together either in sequential or in concurrent fashion in order to manipulate the fiber diameter and density (Section 3.2). Third category involves approaches which use sacrificial elements, in the form of particles or fibers, embedded within the electrospun scaffolds as the porogens (Section 3.3). Fourth category describes methodologies which use specialized fiber collecting devices that aim at obtaining highly porous electrospun mats (Section 3.4). Fifth category describes the approaches involving post-fabrication processing steps aimed at improving the scaffold porosity (Section 3.5). Finally, the sixth category describes all other approaches that do not fall in any of the above categories (Section 3.6). We then put our conclusive remarks in Section 4. To the best of our knowledge, the current review covers the state of the art and the most popular, if not all, methods described in the literature across various indexing platforms, including Scopus, Web of Science, PubMed, and Google Scholar. The review, therefore, is comprehensive and up to date in its scope.

## 2. Electrospun Matrices in Tissue Engineering

**Figure 3:**
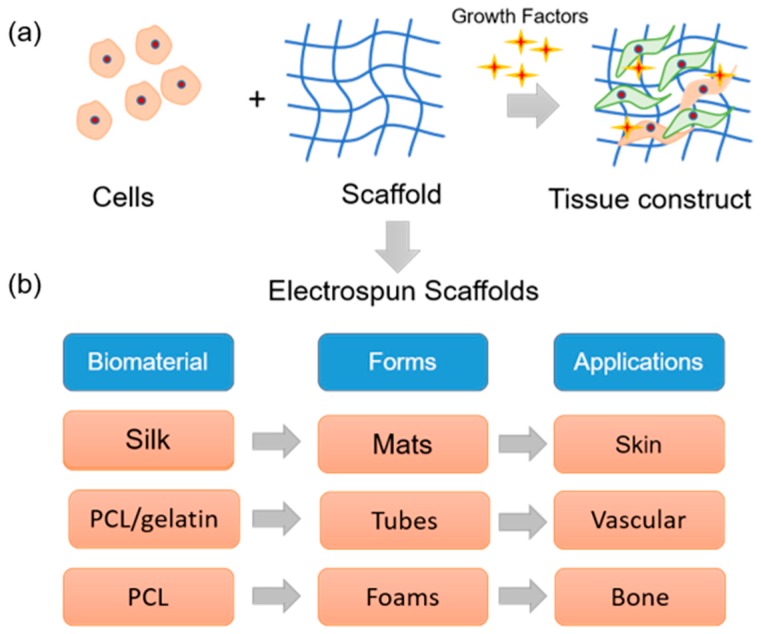
Electrospun matrices in tissue engineering: (**a**) Tissue engineering deals with a combination of cells, natural or synthetic scaffolds, and physiological factors to build a three-dimensional living tissue construct that mimics native tissue. (**b**) By electrospinning technique, different forms of scaffolds can be prepared with natural/synthetic materials that can be used in tissue engineering applications.

Tissue engineering is an emerging interdisciplinary field that aims to regenerate/reconstruct/repair damaged or lost tissues/organs in humans using a combination of cells, scaffolds, and biochemical cues (Figure 3a) [19]. Typically, such bioengineering approaches involve biocompatible and bioresorbable scaffolds that provide environments for cells of interest to grow in a systematic manner and yield a viable and functional new tissues/organs [20]. In native state, the characteristic features of the extracellular matrix (ECM), including the molecules that are associated with viz. collagen, glycosaminoglycans, elastin, etc. and the way in which they are organized, regulate tissue-specific cell response. Generally, the ECM provides structural support and physical environment for cells, provides bioactive cell anchoring moieties such as RGD (Arg-Gly-Asp), offers biomechanical stimuli that induces a cascade of signaling pathways within cells, acts as a reservoir to store and potentiate bioactive molecules such as growth factors, and remodels itself during morphogenesis, homeostasis, and wound healing [21]. In tissue engineering, extensive research on the scaffold aspects so far has suggested that the chemical, physical, structural, and biological properties define the fate of the entire process, and therefore, the prime objective for many researchers is to design an artificial ECM comprised of unique compositional and topographical features that reflect as well as facilitate the functional requirements of a tissue [22].

Considerable efforts have been made to mimic the compositional aspects of the native ECM by exploring natural polymers such as collagen, elastin, silk fibroin, chitosan, etc., synthetic polymers such as polylactic acid (PLA), polycaprolactone (PCL), poly lactic-co-glycolic acid (PLGA), etc., and combinations thereof [23,24]. However, mimicking the intricate network of nano- and submicron- scale fibers in the native ECM posed a challenge. Fortunately, innovations in scaffold fabrication technologies such as electrospinning led to the formation of biomimetic fibrous scaffolds. Being a highly versatile and cost-effective technology, electrospinning has rapidly gained popularity amongst several other techniques [25]. For tissue engineering applications, several polymers including natural, synthetic, and composites have been electrospun to yield scaffolds of various forms (Figure 3b). For instance, Zhang et al. explored an electrospun silk fibroin scaffolds in combination with smooth muscle cells and endothelial cells to develop an artificial vascular graft [26]. Prabhakaran et al. prepared an electrospun PLA-co-PCL/collagen scaffolds and investigated the potential of mesenchymal stem cells for neuronal differentiation toward peripheral nerve repair [27]. Li et al. fabricated random and aligned nanofibrous polyurethane scaffolds and found that human ligament fibroblasts when cultured on aligned matrix yield a higher cell response toward tendon/ligament tissue engineering [28]. Several others demonstrated the feasibility of electrospun scaffolds in engineering various tissues such as cartilage, cornea, liver, etc. [29,30].

Fiber architecture like porosity, pore diameter, and fiber diameter have an effect on cell attachment, spreading, proliferation, and differentiation. Studies showed that nanofibrous chitosan scaffold with a fiber diameter of 300 nm could increase the proliferation and promote the retention of the chondrocyte genotype than a scaffold with a diameter of 1 μm [31]. MC3T3-E1 cells cultured on lower fiber diameter (0.35 µm) PCL electrospun mat upregulated the osteogenic phenotype whereas cells were aligned and highly proliferated on larger fiber diameter mats (6.5 µm) [32]. Researchers also proved that substrate topography influences the immune response activated by macrophages in the early inflammation stage. Proinflammatory molecules secretion by macrophage cells was dependent on Poly(L-Lactic acid) (PLLA) fiber diameter [33]. The analysis of differentially expressed genes of human mesenchymal stem cells (MSCs) cultured on PLLA scaffolds with differing surface topography and chemistry revealed that significant variations in global scale, including the differential expression of hMSC surface markers [34,35]. Culture surfaces have an impact on regulating the expression profile of hypoxy regulatory gene and hypoxy signaling pathway. Fibrous surfaces induced more hypoxia-regulated genes which encoded a complex array of cellular functions than hMSCs cultured on flat surfaces [36]. Pore size is another critical point in scaffold preparation for tissue engineering and regenerative applications. Cell surface interaction can be improved by scaffolds with <1 μm but for a larger pore size around 1–3 µm is required for cell–cell communication and 3–12 µm pore size is required for cell migration through the scaffold [37]. Pore size requirement varies according to tissues and transplanted cell; MSC chondrogenic and osteogenic differentiation demands for 200–400 μm pores [38,39], whereas endothelial cells (10–25 µm), fibroblasts, and nerve cells require smaller pores (20–100 µm) [40,41]. Regeneration of peripheral nerves demanded large pores (100 µm) [42], while smaller pores (<38 μm) are best for microvascular epithelial cells [43]. Researchers also prove that aligned nanofibrous scaffolds augment cardiomyocyte differentiation [44]. In a nutshell, it is possible to fine tune the cell/tissue response by modulating the parameters of the electrospun matrices.

**Figure 4:**
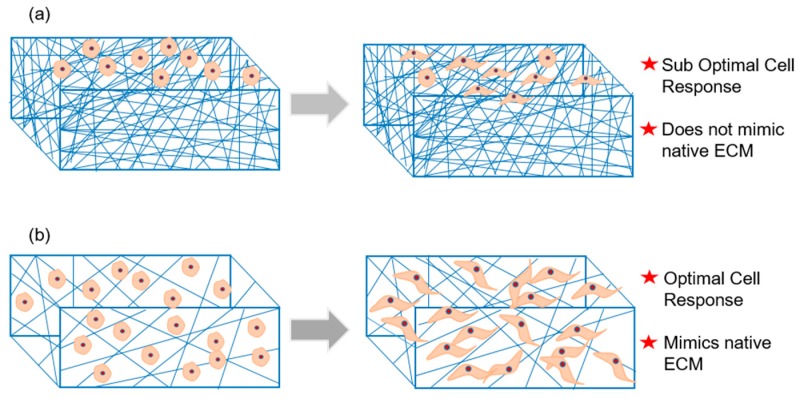
Porosity challenge in electrospun scaffolds: (**a**) Dense packing of electrospun fibers reduces cell infiltration and therefore lead to suboptimal cell response in the conventional setup. (**b**) By any modification in the process setup, any enhancement in the scaffold porosity would enrich cell infiltration and subsequent cell–cell and cell–matrix interactions and therefore would lead to optimal cell response.

Electrospinning technique is not only simple and affordable but also offers control over fiber diameter and arrangement and therefore enables fabrication of scaffolds with variable properties. Besides, electrospun scaffolds are highly recognized for its ultrafine fibrous structure that resembles the nano- and submicron-scale of native ECM, offer a large surface to volume ratio and are exceptionally conducive for cell attachment and growth [45]. However, during typical electrospinning process, fibers are densely deposited on a solid plate in a planar manner with an inter-fiber distance which is much less than a typical cell size. Therefore, upon seeding and culturing, the cells experience a two-dimensional growth profile with minimal infiltration unlike the three-dimensional growth profile of cells in native ECMs (Figure 4) [46]. This means the use of electrospun scaffold in tissue regeneration becomes questionable owing to dense fibrous structure and associated poor cell infiltration. Keeping in view of the correlation between architecture and function that rules typical physiology, it is important in tissue regeneration to fabricate electrospun scaffolds with optimal structural features. To this end, besides merely mimicking native ECMs in their fibrillary structures, significant efforts have been put to achieve spatial characteristics similar to native ECM. The following sections would categorically describe some of the interesting approaches to enhance porosity in electrospun scaffolds.

## 3. Strategies to Enhance Scaffold Porosity

### 3.1. Conventional Electrospinning Methodologies

**Figure 5:**
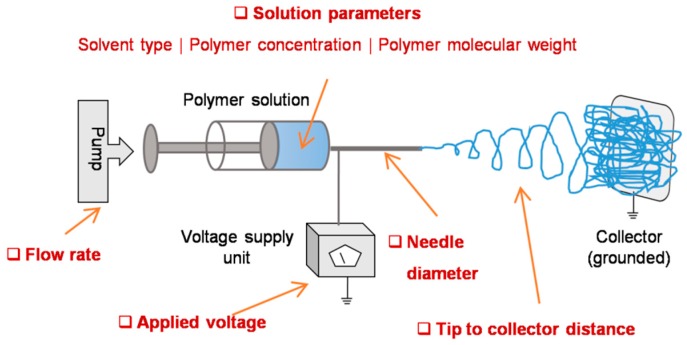
Conventional electrospinning parameters affecting scaffold porosity: solvent properties, polymer properties, processing additives, flow rate, voltage applied, needle gauge, distance from jet evolution point to deposition point, and other parameters such as ambient temperature and humidity are conventionally known to influence the fiber diameter, density and therefore the porosity.

**Figure 6:**
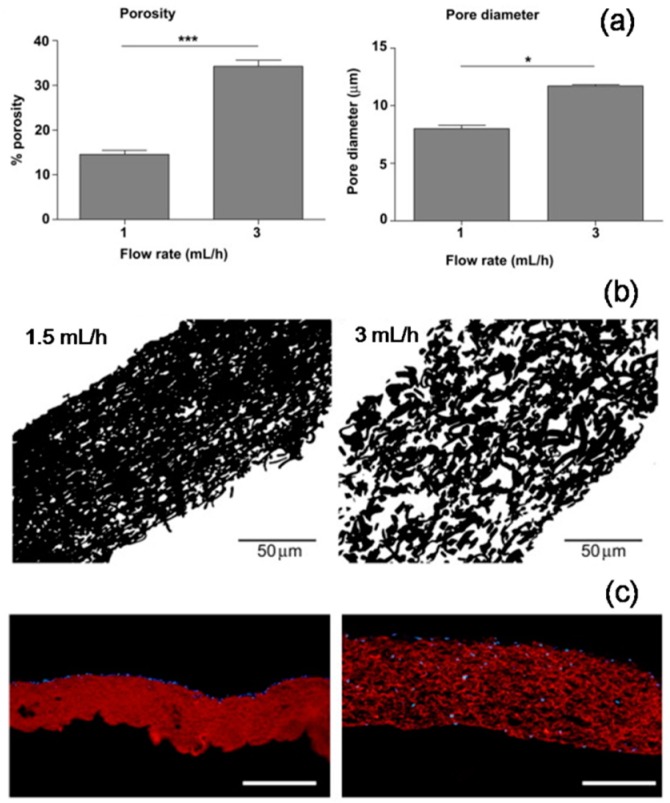
Flow rate induced changes in porosity of electrospun synthetic human elastin scaffold: Increase in flow rate led to an increase in pore diameter and porosity as evident from Image J analysis of SEM pictures (**a**: processed data, **b**: cross-section images). Dermal fibroblast cells largely remained on the surface in case of low porous scaffold, whereas the cells infiltrated deep in case of high porous scaffold (**c**: fluorescence images, blue: DAPI staining, red: scaffold autofluorescence). Adapted with permission from Rnjak-Kovacina et al., 2011 [50] Copyright ©Elsevier 2011.

A straightforward and a simple approach to enhance the pore size in electrospun matrices is by means of systematic optimization of basic electrospinning process parameters that are particularly aimed at alterations in fiber diameter (Figure 5). Eichhorn et al. predicted, through statistical modeling, a direct correlation between the fiber diameter and the pore size in electrospun matrices [47]. Alterations in polymer solution parameters such as solvent type, polymer molecular weight, polymer concentration, etc. and electrospinning process parameters such as applied voltage, flow rate, etc. typically result in variable fiber diameters [48]. For instance, Pham et al. demonstrated that by altering polymer concentration, fiber diameters of PCL electrospun scaffolds can be varied from 4 to 10 μm and subsequently the pore size was enhanced from approximately 20 to 45 μm, respectively [49]. In another example, Rnjak-Kovacina et al. reported that by altering flow rate, synthetic human elastin fiber diameters were varied by 1.4-fold which resulted in a 1.5-fold increase in mean pore size and a 2.3-fold increase in overall porosity (Figure 6) [50]. Several such investigations were reported in order to increase the intra-fiber pore size of electrospun scaffolds made from both synthetic as well as natural polymers. Subsequent effect on cellular infiltration in tissue engineering applications was also established. Sisson et al. prepared electrospun gelatin matrices with small (110 ± 40 nm) and large (600 ± 110 nm) diameter fibers and seeded equal number of osteoblastic MG63 cells [51]. After 2 weeks of culture, confocal imaging showed that cell infiltration was up to a maximum depth of 16 μm in the small-diameter scaffolds, whereas, in the large-diameter scaffolds the infiltration depth was 50 μm. Similarly, Balguid et al. reported that the human venous myofibroblasts infiltration was gradually increased with increasing fiber diameter ranging from 3.4 μm up to 12.1 μm in five scaffold groups [52].

### 3.2. Sequential and Concurrent Electrospinning Approaches

**Figure 7:**
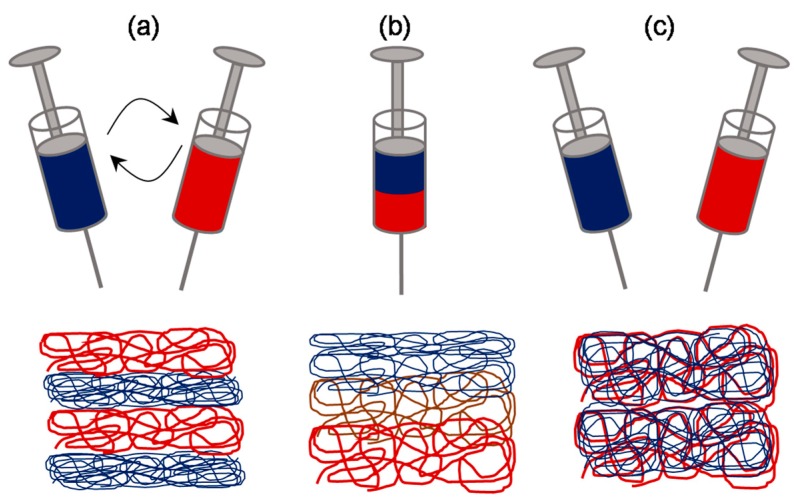
Schematic of sequential and concurrent electrospinning approaches: Two-syringe system can be sequentially used to fabricate layer-by-layer structure of micro- and nanofibers (**a**) or can be concurrently used to fabricate mixed fibrous structure (**c**). Alternatively, single syringe system loaded with two or more portions of variably concentrated polymer solutions can be explored to fabricate a structure with gradient structure (**b**).

By reading through the above examples, it appears that the microfibrous scaffolds are actually superior over the much hyped nanofibrous scaffolds in terms of overall cell infiltration. However, it is important to note that the fiber diameter greatly affects the cell adhesion and thereby the overall cell response. It has been shown previously that the cells seeded onto microfibrous scaffolds flatten and spread similar to those seeded on conventional flat surfaces owing to diameters equal to that of cell size, whereas, cells seeded onto nanofibrous scaffolds attain three-dimensional cell shapes owing to the large number of binding sites presented by the nanofibers [53]. Therefore, efforts were made into making hybrid scaffolds so as to complement the merits of micro- and nanoscale fibers and reduce the limitations thereof (Figure 7). One way to fabricate a scaffold with such hybrid architectural features is by making gradient electrospinning. Recently Kim et al. demonstrated a technique termed as tubing-electrospinning, wherein a series of tubes with PCL solution, ranging from 6% to 20% (w/v) concentration, were combined and subjected to electrospinning without a time lag [54]. This led to the formation of a scaffold with fibers ranging from 0.2 μm to 1.5 μm in a gradient manner. In vitro studies with NIH3T3 cells indicated that cells migrate quickly through the zone having larger void volume created by microscale fibers and thereafter slowly moving through the zone having smaller void volume. Alternatively, bi-modal or two-tier or anisotropic scaffolds were also fabricated by similar sequential electrospinning approaches, wherein micro- and nanofibers were deposited in a layer by layer fashion, typically by sequentially changing syringes with variable concentrations of polymer solution [55,56].

**Figure 8:**
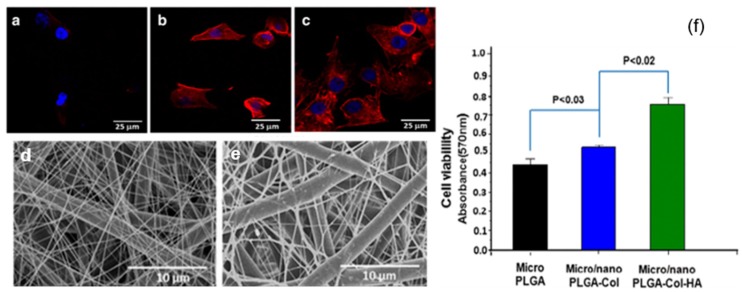
Cell response on a micro/nanofibrous mat prepared by dual electrospinning approach: Actin cytoskeleton staining indicated that MC3T3-E1 cell response was higher on micro/nanofibrous poly lactic-co-glycolic acid (PLGA)-Col (**b**,**d**) and PLGA-Col-HA (**c**,**e**) scaffolds than on microfibrous PLGA scaffold (**a**). The same observation was confirmed quantitatively by cell viability assay (**f**). Adapted from Kwak et al., 2016 [57] © The Authors 2016.

While the scaffolds made with tubing-electrospinning approach are likely to be obtained as one-piece, the rest of sequential electrospinning approaches, due to the time lag, are likely to yield a scaffold with multiple fleeces which may perhaps get disintegrated. In order to overcome this, concurrent or dual/multi-extrusion electrospinning with two or more syringes can be used to produce scaffolds with hybrid architecture in one-piece (Figure 8) [57]. Kim et al. reported such a co-electrospinning strategy by solution electrospinning of silk fibroin in one syringe and melt electrospinning of PCL in another syringe [58]. Process parameters were optimized to yield microfibrous PCL skeleton and nanofibrous reconstituted silk fibroin (RSF) fibers dispersed through it. Keeping microfiber density constant, the ratio of nanofiber density was varied by varying RSF flow rate. In extensive biological studies, it was demonstrated that compared to PCL microfibrous scaffold alone, the in vitro cell response and in vivo tissue response was relatively superior in PCL/RSF hybrid scaffold. However, when two or more syringes are used concurrently there is a potential chance of electrostatic charge repulsions and thereby the polymer jets tend to repel from one another and land at distant areas on the collector. To overcome this issue, Kidoaki et al. reported two strategies [59]. In first case, an insulated object was used, that works as auxiliary electrode, to effectively bring closer the polymer jets and confine the area of deposition to a narrow area of the grounded collector. In second case, a collector with traverse movement was used so as to allow rapid overlapping of micro- and nanofibers coming from different syringes [59].

### 3.3. Approaches Involving Sacrificial Elements

**Figure 9:**
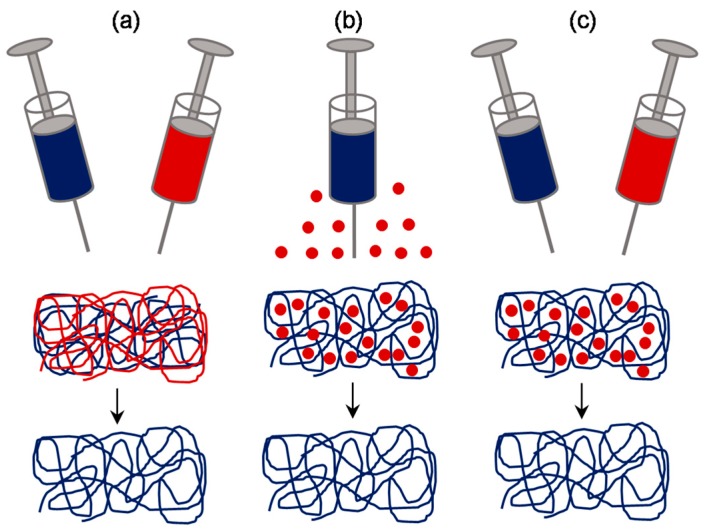
Schematic of approaches involving sacrificial elements: Fibrous sacrificial component could be incorporated by sequential or concurrent electrospinning along with the fibrous component of interest (**a**). Alternatively, particulate sacrificial component could be incorporated either by direct deposition (**b**) or by sequential or concurrent electrospraying (**c**).

Given the large surface area to volume ratio, a scaffold composed of nanoscale fibers was still demanding compared to the one with micro- and nanofibers. Therefore, efforts were put by various researchers in order to enhance the pore size in nanofibrous scaffolds, without compromising on having a microfibrous component within it. One of the most effective ways of achieving this is by fabricating a scaffold composed of elements made of soluble polymer as well as insoluble polymer, followed by selective removal of the soluble polymer elements by rinsing in a suitable dissolution medium (Figure 9). The sacrificial elements could be either fibers or particles incorporated by different means. For instance, Baker et al. demonstrated the fabrication of a scaffold composed of PCL and PEO (Polyethylene oxide) by co-/dual-electrospinning approach [60]. PEO fibers were regarded as the sacrificial component and subsequently removed by rinsing in water. By varying the relative fraction of PEO fibers from 0 to 90%, scaffolds with enhanced pore size and pore area were fabricated. Consequently, cell infiltration efficiency was assessed by culturing MSCs for 3 weeks, wherein, >10% of the total cells infiltrated through the center in scaffold with 60% PEO fraction while no cells infiltrated so in 5% PEO group. Such investigations were carried out to enhance the pore size by several investigators [61], for instance, Voorneveld et al. fabricated PU-based small-diameter vascular grafts [62] and Whited et al. fabricated PLLA scaffolds for bone tissue engineering applications [63]. Further, the relative distribution of sacrificial fibrous component can be made manipulated by following any of sequential electrospinning approach or concurrent electrospinning approach with traverse movement as described in the previous subsection.

**Figure 10:**
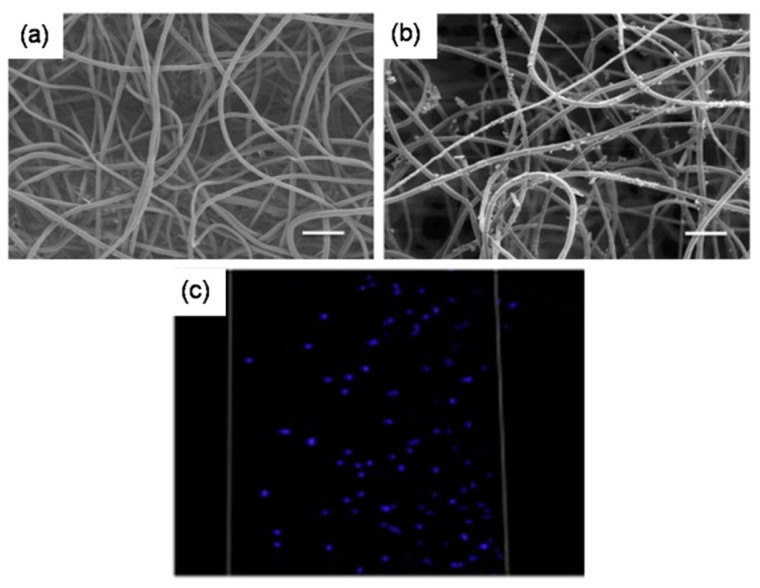
Porosity and cellular infiltration in scaffolds prepared with sacrificial elements: SEM images of PEO/PLLA (75% PEO) before fiber removal (**a**) and after fiber removal (**b**), particulate matter was indicative of biomineralization). Cellular infiltration was complete throughout the scaffold after removal of sacrificial fibers (**c**). Adapted with permission from Whited et al 2011 [63] Copyright ©Elsevier 2011.

Instead of fibers, attempts were made to incorporate sacrificial particles in order to achieve control over pore size and pore volume. One way of incorporating such particles is by concurrent electrospinning and electrospraying [64]. For instance, Wang et al. fabricated RSF electrospun scaffold embedded with PEO microparticles by co-electrospraying [65]. After the removal of PEO particles, the pore size of the scaffold increased from 5.44 ± 2.77 μm to 33.13 ± 8.55 μm. One-week in vitro studies with mouse 3T3 fibroblasts suggested that the cells infiltrated up to 550 μm in the RSF scaffolds with sacrificial PEO microparticles, while those on the control RSF scaffolds remained largely on the surface. In vivo studies in rats revealed that the tissue ingrowth was complete in modified RSF scaffolds than the pristine counterparts. Similar such approaches were investigated and found to be successful in the fabrication of macroporous electrospun scaffolds [66]. Alternatively, a much more straightforward approach was also reported by direct introduction of particles such as salt or sugar. Typically, on top of the scaffold collecting unit, be static or rotating drum, a unit to dispense salt particles in a controlled manner can be kept while performing the electrospinning [67,68]. With this approach, Kim et al. fabricated hyaluronic acid and collagen scaffolds with pores 100–200 μm for bone tissue engineering [69], and Park et al. fabricated RSF scaffold with macropores for skin tissue engineering [70]. In a similar approach, Nam et al. used a special setup wherein a sheath surrounding the needle was created to drop the salt particles of about 100 μm [71] (Figure 10). In another report, Wulkersdorfer et al. sprayed sucrose-ethanol suspension while electrospinning PLGA scaffold and created pores up to 350 μm [72].

### 3.4. Approaches Involving Special Collectors

**Figure 11:**
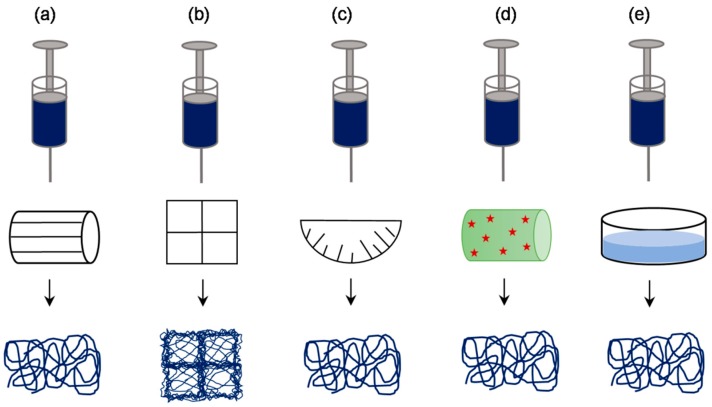
Schematic of approaches involving special collectors: a variety of collectors such as rotating frame cylinder (**a**), patterned grid (**b**), needle-like array dish (**c**), cryogenic plate/mandrel (**d**), and liquid bath collector (**e**), have been used in order to produce electrospun scaffolds with enhanced porosity.

**Figure 12:**
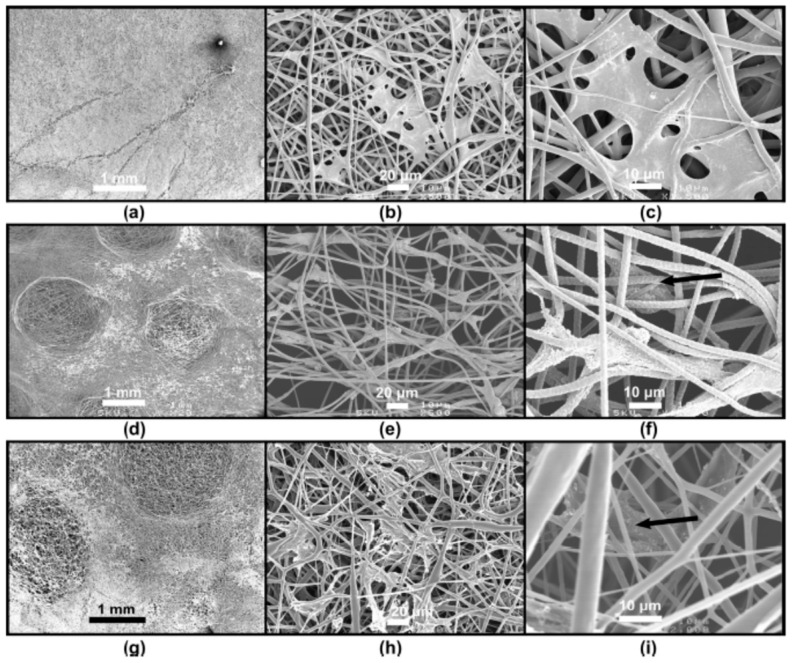
Cell response on electrospun scaffolds prepared by patterned collectors: Compared to the conventional electrospun scaffold (**a**), the scaffolds prepared by patterned collectors were having zones with a lower fiber density of 10-fold increased porosity (**d**,**g**), and as a result, fibroblasts infiltration was relatively enhanced (**b**,**c**: normal, **e**,**f**: and **h**,**i**: patterned). Adapted with permission from Vaquette and Cooper-White, 2011 [76]. Copyright ©Elsevier 2011.

An alternative approach to generate highly porous electrospun scaffolds is by manipulating density and orientation of the deposited fibers. Several researchers have demonstrated the feasibility of this approach using a variety of specialized collectors (Figure 11). One such example was reported by Zhu et al., where the use of a rotating frame cylinder was found to enhance the pore size from 21 μm to 132 μm in PLGA scaffolds for skin tissue engineering applications [46]. Blakeney et al. described the use of a specially crafted spherical dish collector having an array of needle-like probes to create a focused, low-density, uncompressed nanofibrous PCL scaffold [73]. In another interesting approach, McClure et al. replaced the conventional solid rotating mandrel with porous mandrel and purged pressurized air so as to impede fiber deposition in order to create macroporous PCL scaffold [74]. This setup was also used by Yin et al. to fabricate Poly-lactide-co-caprolactone/RSF scaffolds for vascular tissue engineering applications [75]. Vaquette et al. described the use of several patterned collectors such as wire collectors (stainless steel wire meshes having a mesh size of 0.5, 3.3, and 5 mm), round collectors (stainless steel plates in having holes of 0.75, 2, and 3 mm diameter), star collector, ladder collector, and round collectors (Figure 12) [76]. The resultant scaffolds have a high-density and low-density fiber deposition, wherein, the pore size in the latter zones was relatively 10-fold higher. Cell culture studies indicated that the low-density zones allowed fibroblasts up to 250 μm as compared to high-density zones where cells were confined largely to the surface. It was suggested that the size of nonconductive gap in the collector determines the pore size.

In another example, few others explored the use of a low-temperature, cold plate, or cryogenic mandrel under humid conditions, wherein, formation of ice crystal condensing humidity and deposition of polymer fibers occur simultaneously [77]. In the process, the ice crystals get thoroughly embedded within the polymer mesh, and post-fabrication drying leads to template dissolution and formation of pores. The rate of ice crystal formation determines the fiber density and the resulting porosity. This approach was explored by Leong et al. to fabricate highly porous poly(d-lactic acid)scaffold with pores ranging from 900 ± 100 μm^2^ to 5000 ± 2000 μm^2^ depending on the relative humidity used [78,79]. It was claimed that 3T3/NIH fibroblasts infiltrated up to 50 μm depth of the scaffold under in vitro static culture conditions, and subcutaneous implantation in Wistar rats suggested that the cell infiltration was greater than 400 μm. Yet another interesting approach of fabricating a scaffold with low fiber density is by use of a liquid bath or coagulation collector. Popularly called as wet electrospinning, this process requires a non-solvent bath collector, wherein, the properties of the non-solvent bath prevents the dense packing of the fibers and thereby allows the creation of highly porous electrospun scaffolds [80,81,82]. For instance, Majidi et al. fabricated 3D macroporous, alginate/gelatin hydrogel nanofibers and demonstrated that these scaffolds supported proliferation of mesenchymal stem cells over as well as maturation of human induced pluripotent stem cells derived ventricular cardiomyocytes [83].

### 3.5. Approaches Involving Post-Production Processes

**Figure 13:**
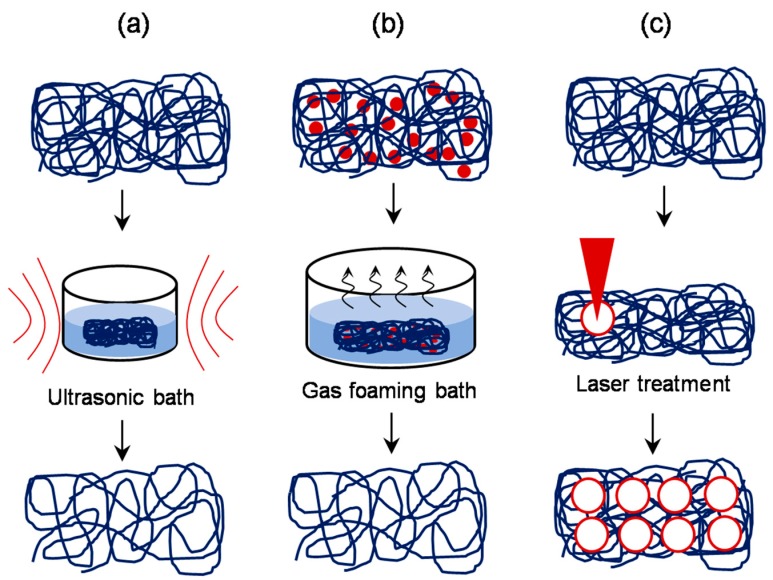
Schematic of approaches involving post-production processing: Controlled exposure of the electrospun scaffold to ultrasonic treatment (**a**), incorporation of sodium borohydride or similar salt in the scaffold and subsequent incubation in appropriate bath to allow gas foaming (**b**), and controlled laser ablation treatment (**c**) are found to be effective in manipulating the pore size after the production.

Although the above described pre- or co-fabrication strategies to enhance the scaffold porosity were found to be largely effective, the requirement of significant changes to the setup is one of the major limitations. Therefore, researchers also attempted to formulate post-production processing of electrospun scaffolds to enhance and control its pore size and porosity (Figure 13). Physical manipulation of electrospun scaffolds by controlled exposure to ultrasonication led to increased porosity and thickness of fibers as well as increased cellular infiltration. Jung et al. explored that ultrasonication of PLLA electrospun nanofibers decrease the spatial density of fibers by mechanical separation via vibrations of ultrasonication [84]. Varying the ultrasonication time and energy, the pore size, porosity, and overall nanofiber scaffold thickness were adjusted NIH3T3 fibroblast cells seeded on top of sonicated electrospun scaffold showed increased cellular penetration up to ~350 μm. Similarly, Gu et al. demonstrated that the ultrasonicated chitosan electrospun nanofibrous mats showed an increase in porosity from 79.9% to 97.2% with one-minute treatment [85]. Aghajanpoor et al. used a combination of sacrificial co-electrospinning with PEO and subsequent ultrasonication of PCL/nanohydroxyapatite electrospun mats which resulted in 1.9 folds increase in pore size; this led to a significant increase in rate of cellular infiltration, cell proliferation, and osteogenic differentiation of hMSCs [86]. Ma et al. reported the use of post-fabrication ultrasonication as a strategy to enhance the porosity in Polyvinylidene fluoride electrospun scaffolds and subsequently to incorporate graphene oxide platelets [87]. It was claimed that pore size and porosity can be tuned by manipulating the ultrasonic energy and exposure time.

**Figure 14:**
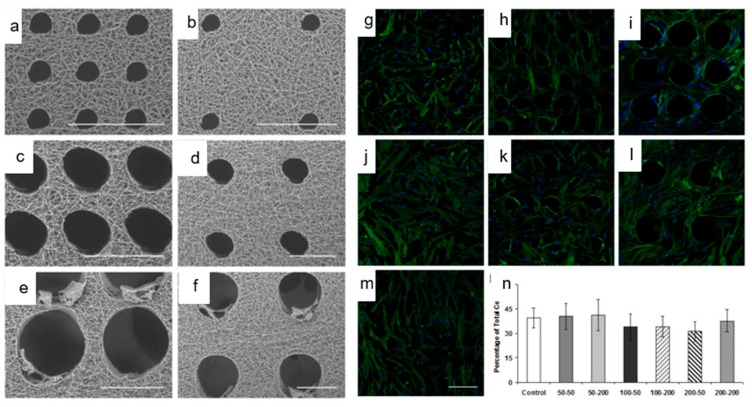
Laser ablation approach to enhance the porosity in electrospun scaffolds: Femtosecond laser ablation approach created pores of defined size without any fiber melting and blockage of porous structure. Adhesion, morphology, and viability of human mesenchymal stem cells (hMSCs) were influenced by pore sizes created by laser ablation (**a**–**f**: SEM images, **g**–**m**: fluorescence images, n: cell proliferation data). Adapted with permission from Lee et al., 2012 [92] Copyright ©Elsevier 2012.

Yet another simple post-production processing approach was reported by Jiang et al. [88], wherein, the technique of gas foaming was employed to enhance the scaffold porosity. Briefly, PCL electrospun scaffolds were suspended in 40 mL freshly prepared sodium borohydride (NaBH_4_) solution under mild shaking (50 rpm). The concentration of NaBH_4_ solution was varied from 0.01 to 1 M and the duration of scaffold treatment was varied from 0 to 24 h. The results revealed that the gross thickness of PCL electrospun scaffolds increased from 1 to 35.6 mm after 24 h treatment in 1 M NaBH_4_ solution, and consequently the scaffold porosity increased to 83.6–99.2%. It was claimed that the porosity can be fine-tuned by changing either the NaBH_4_ concentration or the duration of incubation. In another approach, several researchers explored the use of lasers to create macro-sized pores in the electrospun scaffolds [89,90]. With the laser ablation approach, McCullen et al. created pores of 150, 300, and 600 μm diameter in the electrospun PLA scaffolds [91]. Although the laser ablation resulted in a molten morphology around the pores, *in vitro* cell culture studies with human adipose-derived mesenchymal stem cells suggested that the cells were still able to adhere to the micro-machined scaffolds. Similarly, Lee et al. employed a femtosecond laser system to ablate and create pores of varying size and spacing in electrospun PLLA scaffolds and found that they exhibit significantly enhanced endothelial cell migration and macrophage infiltration compared to the control scaffold *in vivo* (Figure 14) [92].

### 3.6. Other Approaches

**Figure 15:**
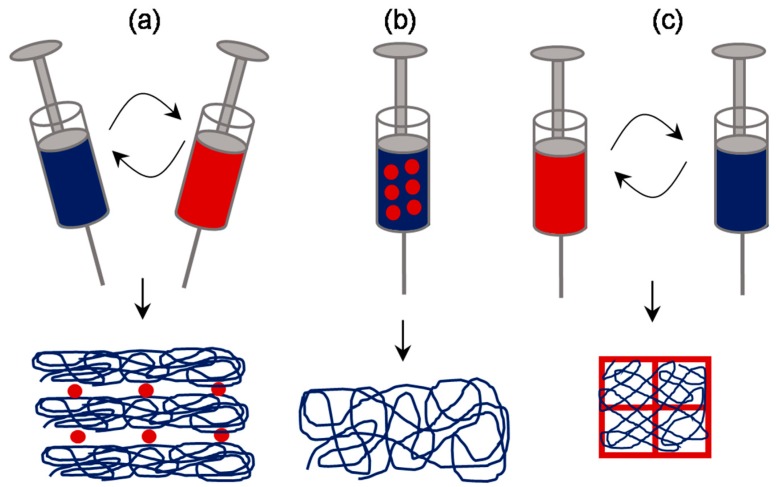
Schematic of electrospinning approaches involving unique concepts: Alternative electrospinning and electrospraying process (**a**), emulsion electrospinning method (**b**), and alternative 3D printing and electrospinning approach (**c**) are reported to yield scaffolds with relatively superior pore properties.

**Figure 16:**
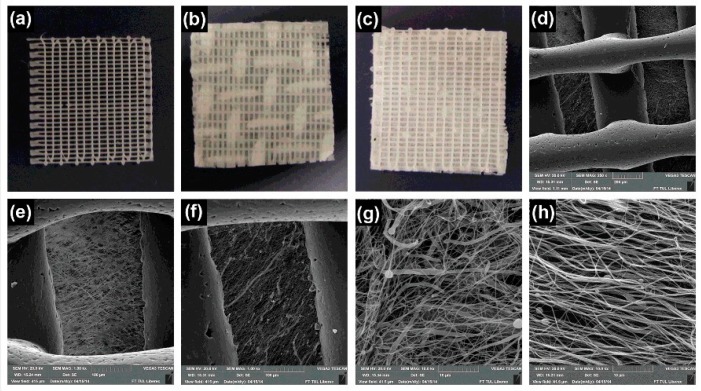
Macro and microporous scaffolds prepared by a combination of electrospinning and 3D printing: Macroscopic images of 3D printed grid without and with patterned and classical electrospun mat (**a**–**c** respectively). SEM images of 3D printed grid with patterned electrospun mat (**d**,**e**—low magnification, **g**—high magnification), and classical electrospun mat (**f**—low magnification, **h**—high magnification). Adapted from Rampichova et al. 2018 [98].

We found few other interesting approaches which could not be classified into any of the above categories (Figure 15). One such example is focused on emulsion electrospinning as reported by Pal et al. [93]. Here, a bilayer scaffold composed of one typical electrospun layer and another highly porous cotton-wool-like 3D layer was made out of PCL–chitosan emulsion. It was claimed that the typical electrospun membrane has a fiber diameter ∼274 nm and pore size ∼1.16 μm whereas the cotton-wool-like layer has a fiber diameter ∼1.62 μm and pore size ∼62 μm. In vivo studies suggested that the scaffold was effective in healing third-degree burn wounds in rat models. Yet another innovative strategy to generate macroporous nanofibrous scaffolds exploits alternative concurrent electrospinning and electrospraying on a micropatterned collector [94]. For instance, Garcia et al. fabricated a composite scaffold with alternating layers of PCL and hydroxyapatite into a honeycomb-like structure with an inner diameter of 160 μm [95]. It was claimed that the porosity of this composite scaffold was capable of providing bone cells with a 3D environment while ensuring the material biomechanical strength. Recently, 3D printing technology has taken the center stage of scaffold fabrication field because of the accuracy and the ability to produce personalized scaffolds [96,97]. Lately, both 3D printing and electrospinning technologies concurrently exploited to fabricate composite scaffolds (Figure 16) [98,99]. For instance, Mellor et al. fabricated a composite scaffold by printing a 2 mm PCL basal section, then placing a PCL electrospun layer and continued printing another 2 mm section on top of the electrospun layer [100]. While Yu et al. prepared a composite scaffold by infusing PCL/gelatin dispersed nanofibers into PCL printing templates [101].

## 4. Conclusive Remarks

Electrospinning has been recognized as a versatile approach in terms of obtaining variously shaped structures out of an array of biomaterials for potential applications in tissue engineering and regenerative medicine. However, the dense packing of the fibers is a major setback wherein the cell response is still suboptimal. To this end, various strategies have been reported in order to enhance the pore size in electrospun scaffolds and thereby to enhance the cell response. A summary highlighting the enhancement in pore properties thereby cell response achieved by various approaches is presented in Table 1. However, as a rule of thumb, there is no one single biomaterial which is universally accepted for all applications, similarly, there is no single fabrication approach which may fulfill all the aspects of a tissue engineering scaffold. The structural properties of tissues in the native state significantly vary from tissue to tissue, and therefore while fabricating a scaffold, an approach that appropriately aims to address tissue-specific requirements should be explored. As described in this review, while there exist approaches that are vast and unique from one another in terms of the way the electrospun scaffold porosity challenge is addressed, we still envisage a greater scope in the development of innovative and alternative approaches.

## Figures and Tables

**Table 1:** A summary presenting various approaches in enhancing pore size and thereby cell response in electrospun scaffolds for tissue engineering applications.

Strategy	Material	Pore Properties	Cell Infiltration	Reference
Conventional electrospinning	Gelatin	Variation in fiber diameter from 110 nm to 600 nm	Osteoblastic MG63 cell infiltration enhanced from 16 µm to 50 µm depth	Sisson et al. [51]
Sequential electrospinning	PCL	Variation in fiber diameter from 200 nm to 1.5 μm in a gradient manner	NIH3T3 cells infiltration quickly through the microscale fibrous zone then slowed down through the nanoscale fibrous zone.	Kim et al. [54]
Concurrent electrospinning	PLGA, PLGA-Collagen and PLGA-Collagen-Hydroxy apatite	Variation in fiber diameter and packing density from microscale to micro/nanoscale.	MC3T3-E1 cell viability was increased by 2-fold from microscale scaffold to micro/nanoscale scaffold	Kwak et al. [57]
Electrospinning with sacrificial elements	Silk Fibroin and PEO	Variation in pore size 5.44 μm to 33.13 μm	Cells infiltration was enhanced up to 550 μm depth	Wang et al. [65]
Electrospinning on rotating collectors	PLGA	Variation in pore size from 21 μm to 132 μm	Cell infiltration was enhanced >100 μm depth	Zhu et al. [46]
Electrospinning on patterned collectors	PCL	Variation in pore size by about 10 folds	Cells infiltration was enhanced up to 250 μm depth	Vaquette et al. [76]
Cryogenic electrospinning	PLA	Variation in pore volume from 900 μm^2^ to 5000 μm^2^	cell infiltration was greater than 400 μm depth in vivo	Leong et al. [78,79]
Post-electrospinning ultrasonication	Chitosan	Variation in porosity from 79% to 97%	Cell infiltration was enhanced by 1.4-fold	Gu et al. [85]
Post-production electrospinning – gas foaming	PCL	Variation in porosity from 83.6% to 99.2%	Cells infiltration was seen only in gas foamed scaffold	Jiang et al. [88]
Post-production laser ablation	PLA	Variation in pore size from 21 to 130 um	Enhanced cell migration and infiltration through ablated pores	Lee et al. [92]
Emulsion electrospinning	PCL and chitosan	Variation in pore size from 1 μm to 62 μm	Enhanced cell proliferation was seen within 3 weeks	Pal et al. [93]
3D printing co-electrospinning	PCL	Variation in fiber diameter and packing density	Enhanced cell proliferation was seen in 3D/Espun scaffold than on 3D alone and Espun alone scaffolds	Mellor et al. [100]
